# Differential gene expression in leaf tissues between mutant and wild-type genotypes response to late leaf spot in peanut (*Arachis hypogaea* L.)

**DOI:** 10.1371/journal.pone.0183428

**Published:** 2017-08-25

**Authors:** Suoyi Han, Hua Liu, Mei Yan, Feiyan Qi, Yaqi Wang, Ziqi Sun, Bingyan Huang, Wenzhao Dong, Fengshou Tang, Xinyou Zhang, Guohao He

**Affiliations:** 1 Industrial Crops Research Institute, Henan Academy of Agricultural Sciences, Zhengzhou, Henan, China; 2 Department of Agricultural and Environmental Sciences, College of Agriculture, Environment and Nutrition Sciences, Tuskegee University, Tuskegee, Alabama, United States of America; 3 Henan Provincial Key Laboratory for Oil Crops Improvement, Zhengzhou, Henan, China; 4 Key Laboratory of Oil Crops in Huanghuaihai Plains, Ministry of Agriculture, Zhengzhou, Henan, China; Texas Tech University, UNITED STATES

## Abstract

Late leaf spot (LLS) is a major foliar disease in peanut (*A*. *hypogaea* L.) worldwide, causing significant losses of potential yield in the absence of fungicide applications. Mutants are important materials to study the function of disease-related genes. In this study, the mutant line M14 was derived from cultivar Yuanza 9102 treated with EMS. Yuanza 9102 was selected from an interspecific cross of cultivar Baisha 1016 with *A*. *diogoi*, and is resistant to several fungal diseases. By contrast, the M14 was highly susceptible to late leaf spot. RNA-Seq analysis in the leaf tissues of the M14 and its wild type Yuanza 9102 under pathogen challenge showed 2219 differentially expressed genes including1317 up-regulated genes and 902 down-regulated genes. Of these genes, 1541, 1988, 1344, 643 and 533 unigenes were obtained and annotated by public protein databases of SwissPort, TrEMBL, gene ontology (GO), KEGG and clusters of orthologous groups (COG), respectively. Differentially expressed genes (DEGs) showed that expression of inducible pathogenesis-related (PR) proteins was significantly up-regulated; in the meantime DEGs related to photosynthesis were down-regulated in the susceptible M14 in comparison to the resistant WT. Moreover, the up-regulated WRKY transcription factors and down-regulated plant hormones related to plant growth were detected in the M14. The results suggest that down-regulated chloroplast genes, up-regulated WRKY transcription factors, and depressed plant hormones related to plant growth in the M14 might coordinately render the susceptibility though there was a significant high level of PRs. Those negative effectors might be triggered in the susceptible plant by fungal infection and resulted in reduction of photosynthesis and phytohormones and led to symptom formation.

## Introduction

The cultivated peanut (*Arachis hypogaea* L.) has been known as one of the most important oil seed crops worldwide, providing high quality edible oil and high protein content in the seed. However, plants are often exposed to biotic stresses mainly caused by bacteria, fungi, viruses, nematodes and insects. In peanut, late leaf spot (LLS) caused by *Phaeoisariopsis personata* (Berk. and M. A. Curtis) is the serious foliar disease causing significant peanut yield losses [1], ranging from 10%—80% with inadequate control measures [2–4]. Although multiple applications of fungicides can effectively control of leaf spot disease, fungicides spray could cause environmental pollution and increase production costs [5]. The most economical and environment-friendly management strategy to control LLS disease is to develop and utilize resistant varieties in the crop production [6–8]. Many efforts have been made to identify sources of resistance by extensive screening germplasm [9,10]. Several diploid wild species and tetraploid cultivars have showed high levels of resistance to LLS [11,12]. Utilization of these resistance sources has developed superior varieties resistant to LLS, but conventional resistance breeding is time-consuming [13]. Despite some of DNA markers have been identified as the potential link to the resistance [14,15], they could not be efficiently used in marker-assisted selection because the mechanism of resistance to LLS is complex and polygenic nature and probably controlled by several recessive genes [16,17]. Furthermore, additive genetic variance seems to contribute predominantly to the resistance [18]. To better understanding of molecular mechanism governing LLS resistance, genome-wide transcriptom profiles are necessary.

Next-generation RNA-sequencing (RNA-seq) is now well-established as a versatile platform with applications in many fields of plant biology research [19]. This method detects the expressed sequences in specific tissue at a specific time, and thus can be used to characterize differential expression or tissue-specific transcripts to study plant responses to abiotic and biotic stresses [20]. In the cultivated peanut, a primary screening of differentially expressed genes using cDNA microarray have identified several differentially expressed genes in resistant and susceptible genotypes, but failed to identify the specific genes involved in the resistance to LLS [4]. Recently, Kumar and Kirti (2015) investigated the molecular responses to LLS pathogen using cDNA-AFLP and identified 233 reliable differentially expressed genes in diploid wild species, *Arachis diogoi*, that has a high level of resistance to LLS [11]. They have identified nineteen transcript-derived fragments (TDFs) associated with defense, signal transduction and metabolism in resistant genotypes in comparison with susceptible genotype.

The aim of this study was to investigate and compare transcriptomes between resistant and susceptible genotypes upon pathogen challenge by using RNA-seq technology. The specific objectives were to (1) identify differentially expressed genes between two genotypes; (2) provide specific genes involving in the interaction between peanut host and pathogen *P*. *personata*.

## Materials and methods

### Plant growth and tissue collections

**Fig 1:**
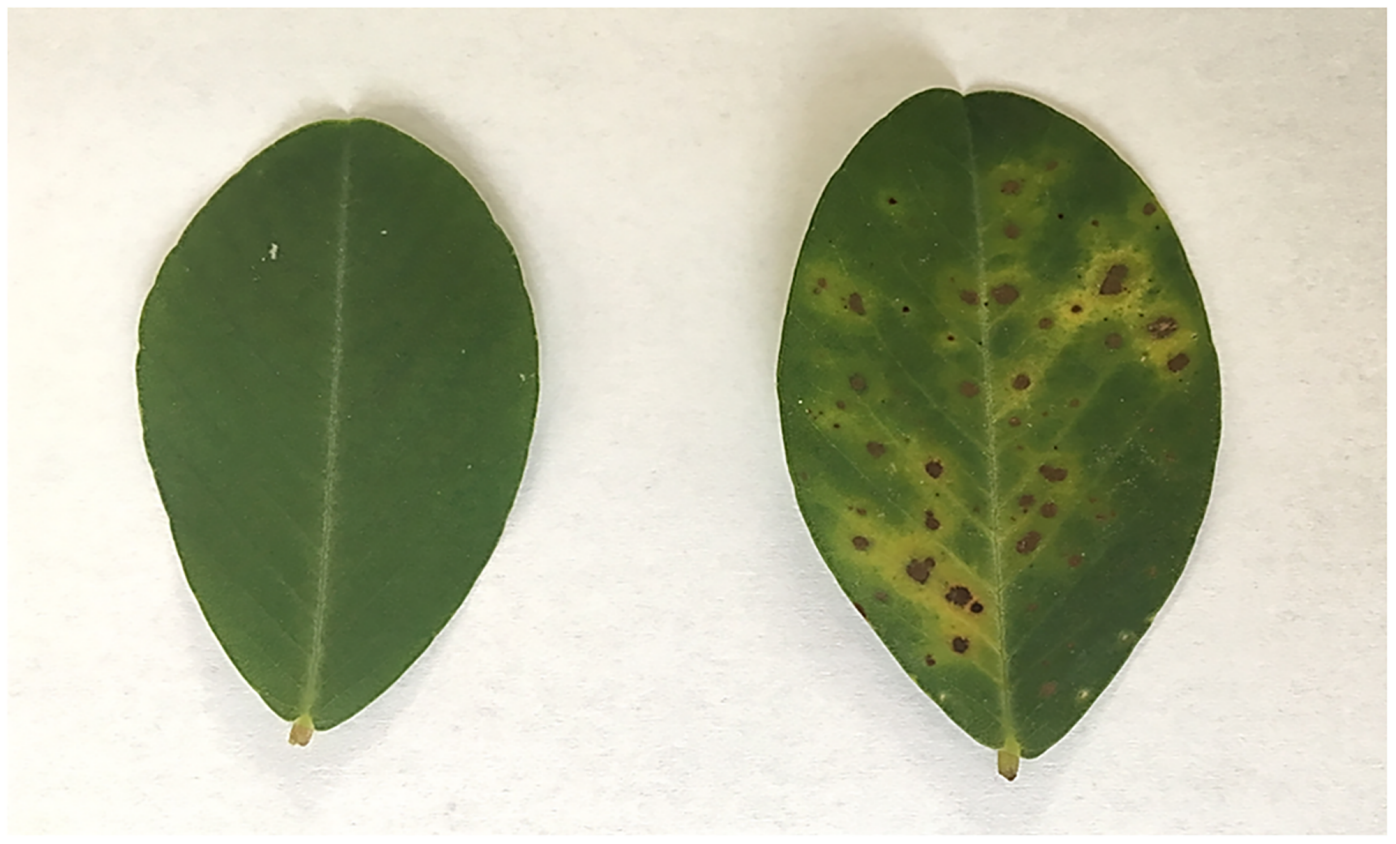
Leaves of resistant Yuanza 9102 (WT) and susceptible mutant (M14) response to late leaf spot infection.

The peanut mutant M14 was derived from the widely grown peanut cultivar Yuanza 9102 by chemical mutagen ethyl methanesulfonate (EMS). The cultivar Yuanza 9102 was developed from an interspecific cross of cultivar Baisha 1016 with diploidy species *A*. *diogoi* through several generation selections based on resistance trait. Yuanza 9102 is highly resistant to several diseases including leaf spot. After treating Yuanza 9102 with EMS, one of mutant lines, M14, was found highly susceptible to LLS. Leaf spot symptom began to appear in the mutant M14 about 30–35 days after germination in the field observed for several years under different climate conditions. The mutant M14 could eventually reach the highest level of 1–9 disease index (Fig 1). Two genotypes were excellent materials to study differentially expressed genes of resistance to LLS because their contrast of phenotypes could be studied on the basis of similar genetic background. The mutant M14 and its wild-type (WT) were planted at the Yuanyang Experimental Station, Henan Academy of Agricultural Sciences, China. Leaf tissues were collected from six plants and were pooled for RNA extraction in each of susceptible (M14) and resistant (WT) Yuanza 9102 genotypes at 30 days after germination before symptoms appeared.

### RNA isolation and gene expression profiling analysis

Leaves were rapidly frozen in liquid nitrogen and were ground to a powder using a mortar and pestle. Total RNA was extracted from leaf tissues using E.Z.N.A. Plant RNA Kit (Omega Bio-tek, USA, R6827-01) according to the manufacturer’s instructions, and treated with RNAase-free DNAase. The quantity and quality of RNA samples were determined by the use of Agilent 2100 bioanalyzer and a Nanodrop ND 1000 spectrophotometer (NanodropTechnologies Inc., Rockland, DE, USA).

To enrich the sequence depth of mRNA, NEBNext Ploy (A) mRNA Magnetic Isolation Module (NEB, E7490) was used to remove rRNA and tRNA. The enriched mRNA samples were subjected to be fragmented and converted into single-stranded cDNA using random hexamer priming. The second strand cDNA synthesis was subsequently performed using DNA Polymerase I and RNase H. Libraries of cDNA were constructed using NEBNext mRNA Library Prep Master Mix Set for Illumina (NEB, E6110) and NEBNext Multiplex Oligos for Illumina (NEB, E7500) following the manufacturer’s recommendations. Fragments of cDNA were visualized in 1.8% agarose gel and suitable length fragments were used to construct final cDNA libraries for paired-end sequencing. Transcriptome sequencing using the Illumina Cluster Station and Illumina Genome Analyzer HiSeq 2000 sequencing platform were performed by the IlluminaPipeline at the Biomarker Technologies Co., Ltd, Beijing, China.

### Raw sequences assembly and data analysis

Before raw sequences were assembled, the parameters of average Q20 percentage (the ratio of the sum of nucleotides with quality value ≥20 and the number of nucleotides) and Q30 percentage (the percentage of number of nucleotides with quality value ≥30 in total number of nucleotides) were used to assess the sequencing quality, in which Q30 above 80% was considered as reliable and good quality sequences. After removing the low quality nucleotides, the raw sequences from two genotypes were separately assembled to form longer contiguous sequences (contigs) using Trinity software [21]. Based on the similarity of paired-end sequence information and contig, contigs were clustered and assembled into transcripts. From each cluster, the longest transcript was subsequently defined as unigenes. The putative open reading frames (ORFs) of unigenes were predicted using EMBOSS/Getorf software [22].

### Functional annotation and classification of the unigenes

To obtain functional annotation of unigenes, BLAST alignment was performed using publically available protein databases including NCBI non-redundant protein (NR), Swiss-Prot protein, Translated European Molecular Biology Laboratory (TrEMBL), Clusters of Orthologous Groups (COGs), Gene Ontology (GO), and Kyoto Encyclopedia of Genes and Genomes (KEGG). All unigenes were assigned GO annotations using Blast2GO (http://www.blast2go.org).

### Differentially expressed gene analysis

Gene expression of all unigenes in M14 and WT were estimated by calculating read density as ‘reads per kilobase of exon model per million mapped reads’ (RPKM) [23]. DESeq software was used to identify the DEGs based on a statistical analysis of large matrices of multi-conditional expression data [24]. A threshold for false discovery rate (FDR) of <0.001 and an absolute value of log2FC were used to determine significant differences in gene expression.

### Quantitative RT-PCR validation

Six functionally important and representative genes were selected for validation of gene regulation using quantitative RT-PCR. Gene-specific primers were designed for each gene, and the ADH3 gene was used as the internal control [25]. Total RNAs of peanut leaves were extracted using E.Z.N.A. Plant RNA Kit (Omega Bio-tek, USA, R6827-01). Samples of 1 μg RNA were used to synthesize the first-strand cDNA using a reverse-transcription system (Promega, USA, A3500) according to the manufacturer’s instructions. Quantitative real-time PCR was performed on the Eppendorf Realplex (4) PCR machine (Eppendorf, Hamburg, Germany) using a reaction volume of 20 μl contained 2× SYBR Green PCR RealMaster Mix (TianGen, FP202-02), forward and reverse primers, and cDNA template (diluted 1:40). All samples were run in duplicate. The real-time PCR reaction was run for one cycle (94°C for 3 min) followed by 40 cycles (94°C for 15 s, 59°C for 20 s, 68°C for 40 s) and fluorescence emissions were measured after each of the repetitive cycles. The results were obtained based on the average of three parallel experiments.

## Results

Phenotypic traits were observed different between susceptible M14 and resistant WT genotypes after infection by pathogens. We have statistically tested three phenotypic traits related with plant growth, i.e. main stem height, 100 pod weight, and 100 seed weight. Three traits were significant different in M14 from those in WT (S1 Fig). These differences indicated that plant growth in the M14 was more depressed by pathogen infection than in the WT.

### Transcriptome sequencing and de novo assembly

To elucidate genome-level responses to LLS disease, transcriptome profiling was performed to identify genes that are differentially expressed between resistant WT and susceptible M14 genotypes. A total of 26,761,569 and 31,198,410 raw reads were obtained from WT and M14 genotype’s cDNA libraries, respectively, encompassing ~11.7 Gb of sequencing data (Table 1). All raw data were deposited in the National Center for Biotechnology Information (NCBI) as accession SRX3088919 and SRX3088920 (https://www.ncbi.nlm.nih.gov/Traces/study/?acc=SRP115293). Statistical evaluation of sequence quality showed 100% sequences with quality value ≥20 and 81.08% sequences with quality value ≥30, indicating good quality and reliable sequences. Trinity software was used to obtain contigs by assembly sequence overlap. Based on the similarity clustering of contigs and information on contig paired-end sequences between the local and assembled transcripts, the longest transcripts from the local were considered as unigene, resulting in total number of 169,246 transcripts and 63,337 unigenes with a N50 of 1,980 transcripts and 1,468 unigenes, respectively (Table 2).

**Table 1:** Statistical evaluation of the sample sequencing data.

Samples	Total reads	Total nucleotides (bp)	Average Q20 percentage	GC percentage	Q30 percentage
WT	26,761,569	5,405,452,644	100%	45.32%	81.15%
M14	31,198,410	6,301,622,837	100%	45.23%	81.08%
Total	57,959,979	11,707,075,481	100%	45.28%	81.12%

**Table 2:** Summary of transcriptome assembly.

Length range	Contigs	Transcripts	Unigenes
200–300	2,986,855(98.08%)	27,179(16.06%)	20,376(32.17%)
300–500	25,036(0.82%)	27,924(16.50%)	17,303(27.32%)
500–1000	17,369(0.57%)	33,670(19.89%)	11,343(17.91%)
1000–2000	10,470(0.34%)	45,329(26.78%)	8,151(12.87%)
2000+	5,593(0.18%)	35,144(20.77%)	6,164(9.73%)
Total number	3,045,323	169,246	63,337
Total length	206,440,261	213,850,833	49,792,597
N50 length	61	1,980	1,468
Mean length	67.79	1263.55	786.15

### Homologous species distribution

**Fig 2:**
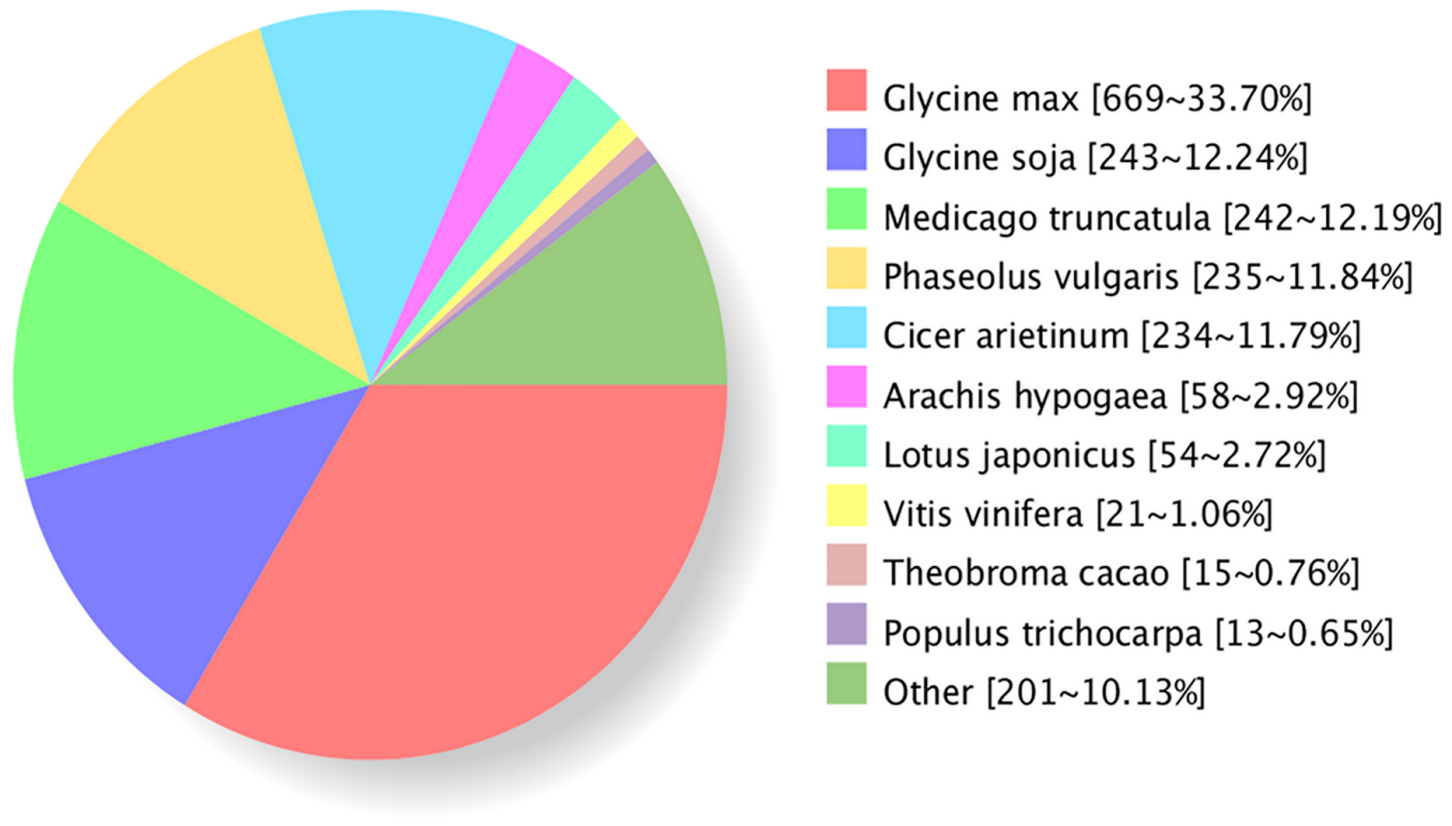
Homologous species distribution of peanut unigenes.

To search for homologous proteins for unigenes, BLASTX was used against the non-redundant nr database with an e-value cut-off of 1.0e^-5^ in the GenBank. Most of unigenes (87.4%) had significant homology with those proteins from legume species (Fig 2). The homologous species distribution showed that 45.94% of unigenes had homology to sequences from *Glycine max* and *Glycine soja*, followed by (12.19%) *Medicago truncatula*, (11.84%) *Phaseolus vulgaris*, (11.79%) *Cicer arietinum*, (2.92%) *Arachis hypogaea*, and (2.72%) *Lotus japonicas*. The most BLAST hits belonged to the legume family, suggesting that unigenes obtained in this study were appropriately annotated. But unigenes had fewer peanut sequence hits; it may indicate that there were fewer peanut sequences in the databases compared to other legume species, such as soybean.

### Identification of DEGs

**Fig 3:**
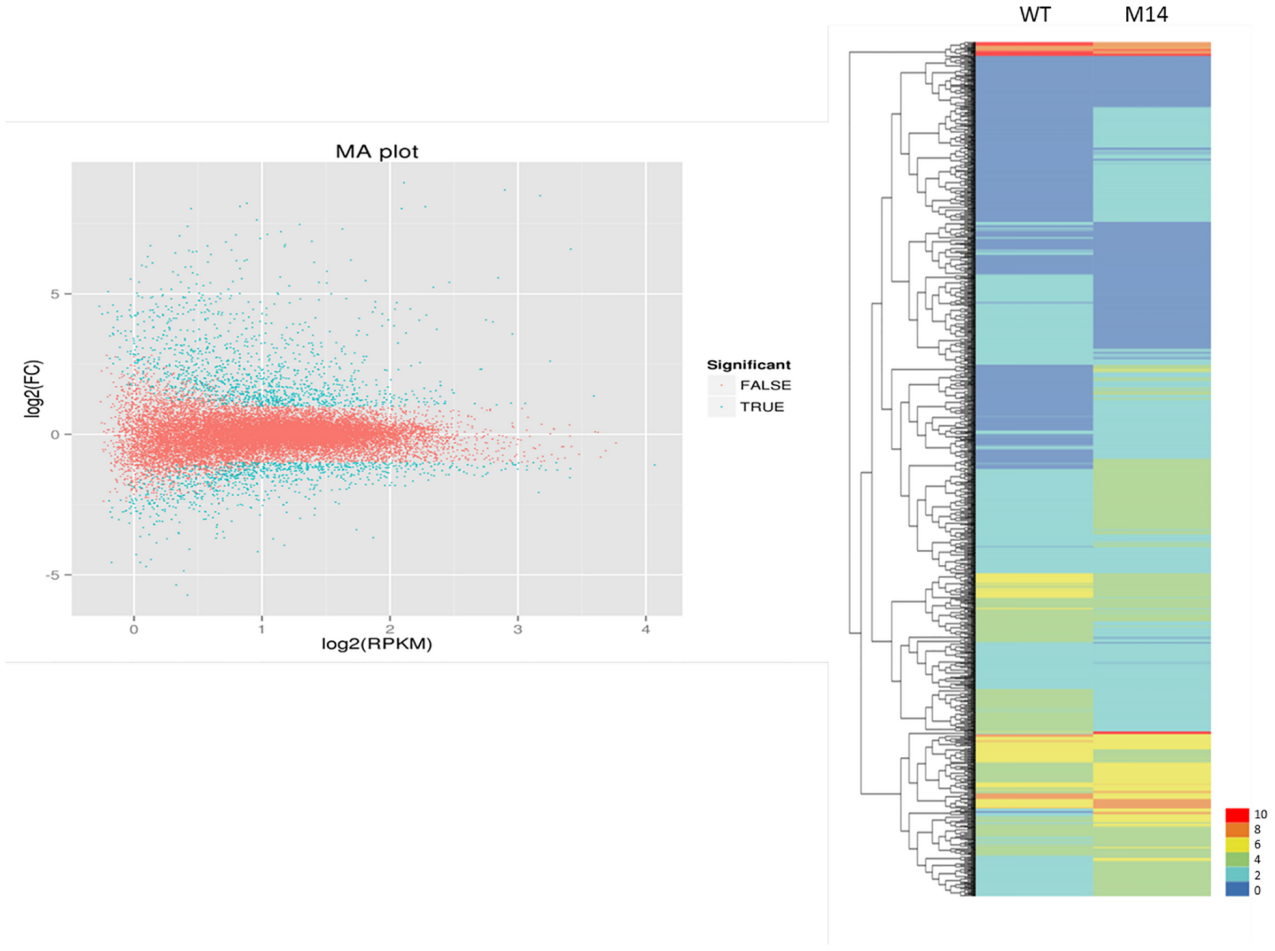
(A) The scatter plot of differentially expressed genes. Blue points represent differentially expressed genes and red points refer to no differential expression. (B) Differential expression levels between two genotypes. The higher score with different color represents the higher level of expression.

To identify the differentially expressed genes (DEGs) between resistant WT and susceptible M14, the reads data from both genotypes were separately mapped to the combined unigene sequences from *de novo* assembled contigs. A total of 63,337 unigenes showed detectable levels of expression in M14 and WT. All unigenes were analyzed for differences in their level of expression in the two genotypes using DESeq software (Fig 3A and 3B). There were 2219 differentially expressed genes, in which 902 DEGs had lower expression levels, while 1317 DEGs were activated in the M14 mutant compared to the resistant WT. In addition, DEGs were mapped to peanut reference genomes (https://www.peanutbase.org) showing that 65.8% of the DEGs matched *A*. *duranensis*, and 34.2% matched *A*. *ipaensis*. All 2219 differentially expressed genes were submitted to the GenBank (http://www.ncbi.nlm.nih.gov/bioproject/375971).

### Functional annotation of DEGs

**Fig 4:**
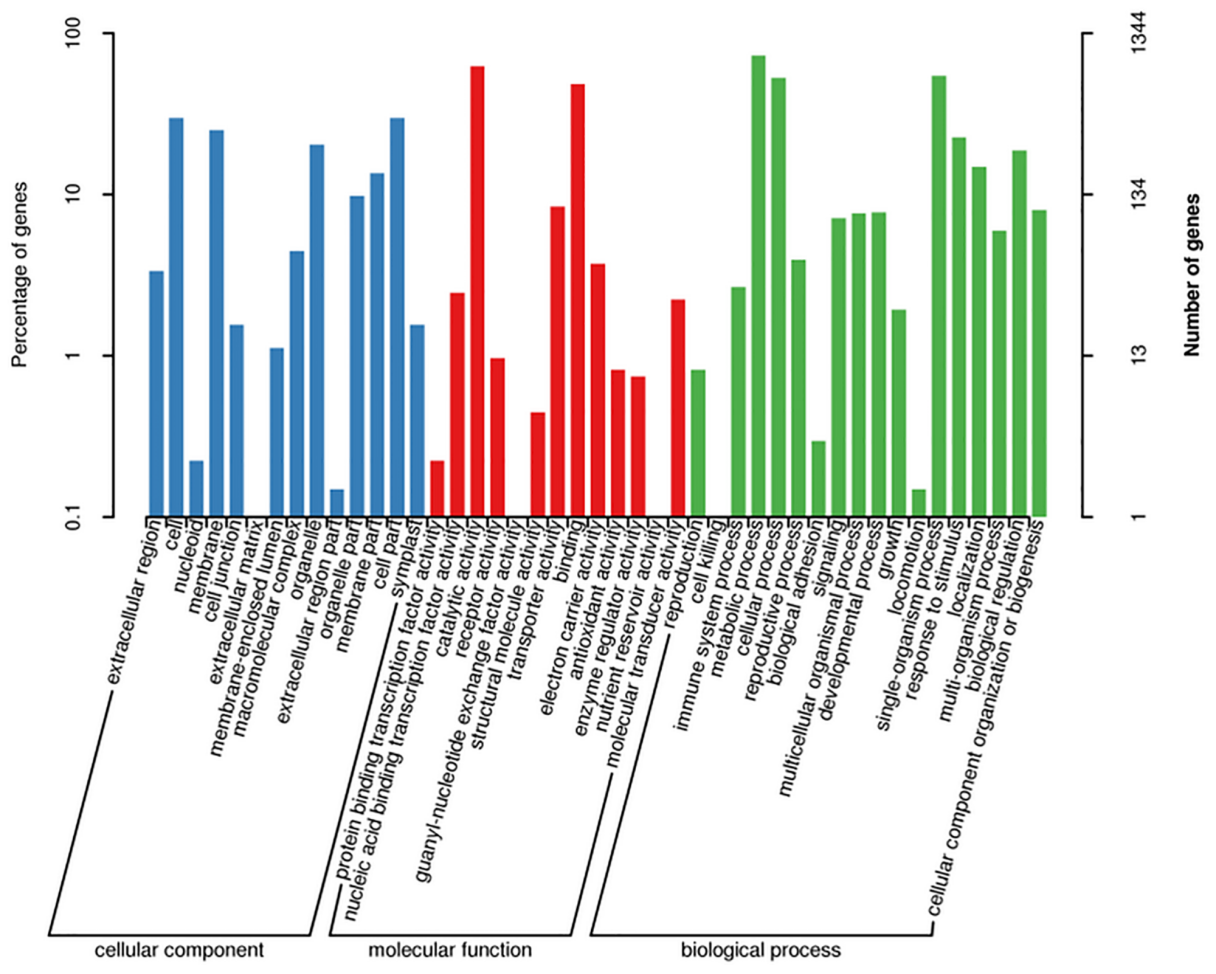
Functional annotation of assembled sequences based on gene ontology (GO) categorization.

For functional categorization of the DEGs, Gene Ontology (GO) analysis was utilized to classify the DEGs using the Blast2GO program. Among 2219 DEGs, 222 DEGs could not be assigned in any GO classes and not annotated. The rest of 1997 DEGs were assigned to three gene ontology classes: biological process (49.5%), cellular component (15.8%) and molecular function (34.7%), with 45 functional terms (Fig 4). In each GO class, the number of up-regulated DEGs was more than those of down-regulated in the transcriptome of M14. Within each of three categories of GO classification, DEGs involved in “cellular process” (1,425), “metabolic process” (1,415), and “response to stimulus” (1,273) were predominant among biological processes; “cell part” (1,406), “cell” (1,375), and “membrane” (1,197) among cellular components; and “catalytic activity” (1,049) and “binding” (1,037) among molecular functions.

**Fig 5:**
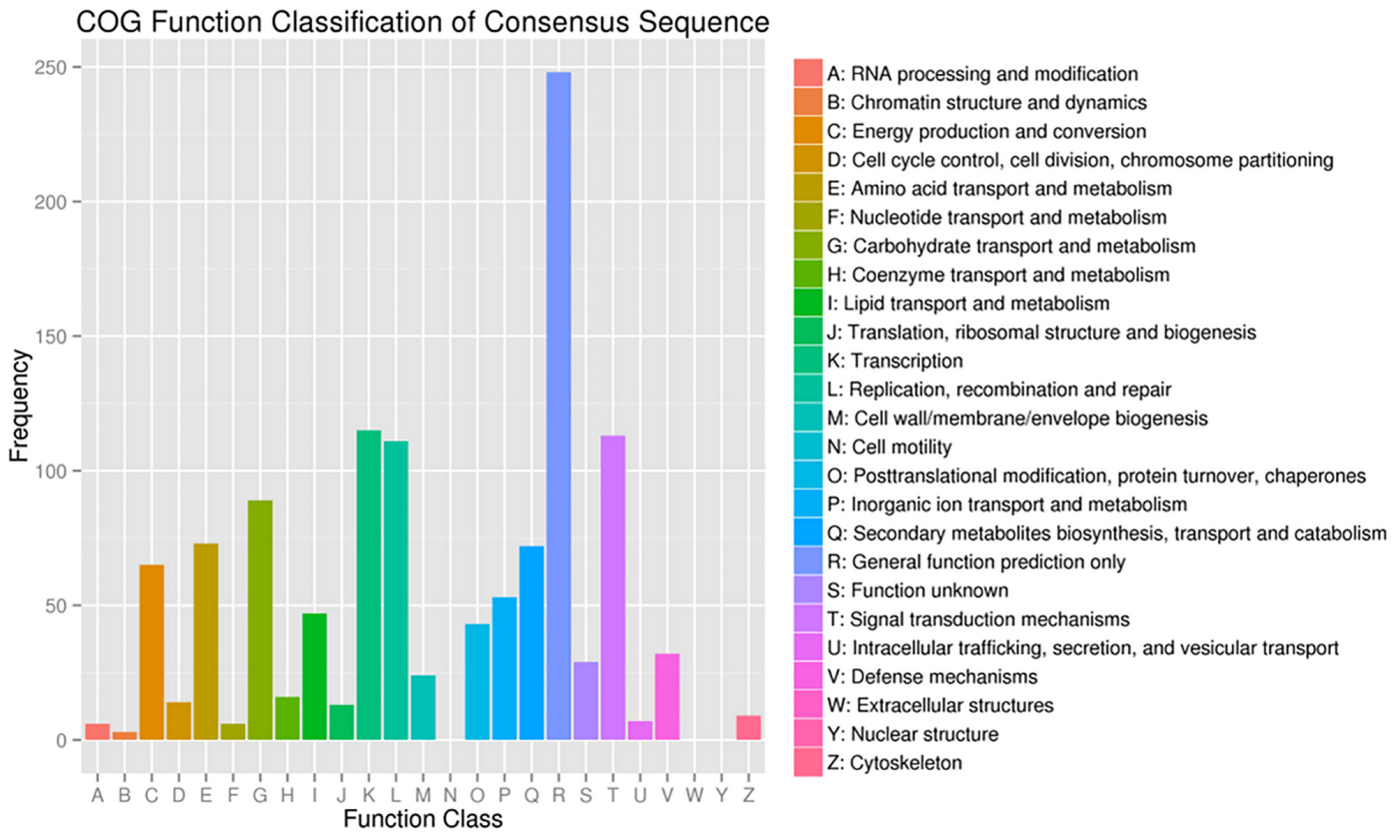
Cluster of orthologous groups (COG) classification.

Based on the conserved domain-based alignment, 2219 DEGs were further compared against Cluster of orthologous groups (COG) database and 533 COG-annotated putative proteins were functionally classified into 20 function categories (Fig 5). Among the 20 COG categories, the cluster for “General function prediction only” (98, 18.4%) represented the largest group, followed by “Carbohydrate transport and metabolism” (57, 10.7%), “Energy production and conversion” (49, 9.2%), “Amino acid transport and metabolism” (49, 9.2%), “Replication, recombination and repair” (14, 2.6%), “Signal transduction mechanisms” (13, 2.4%), and “Transcription” (11, 2.1%). The categories of “extracellular structures” and “nuclear structures” were the smallest groups.

**Fig 6:**
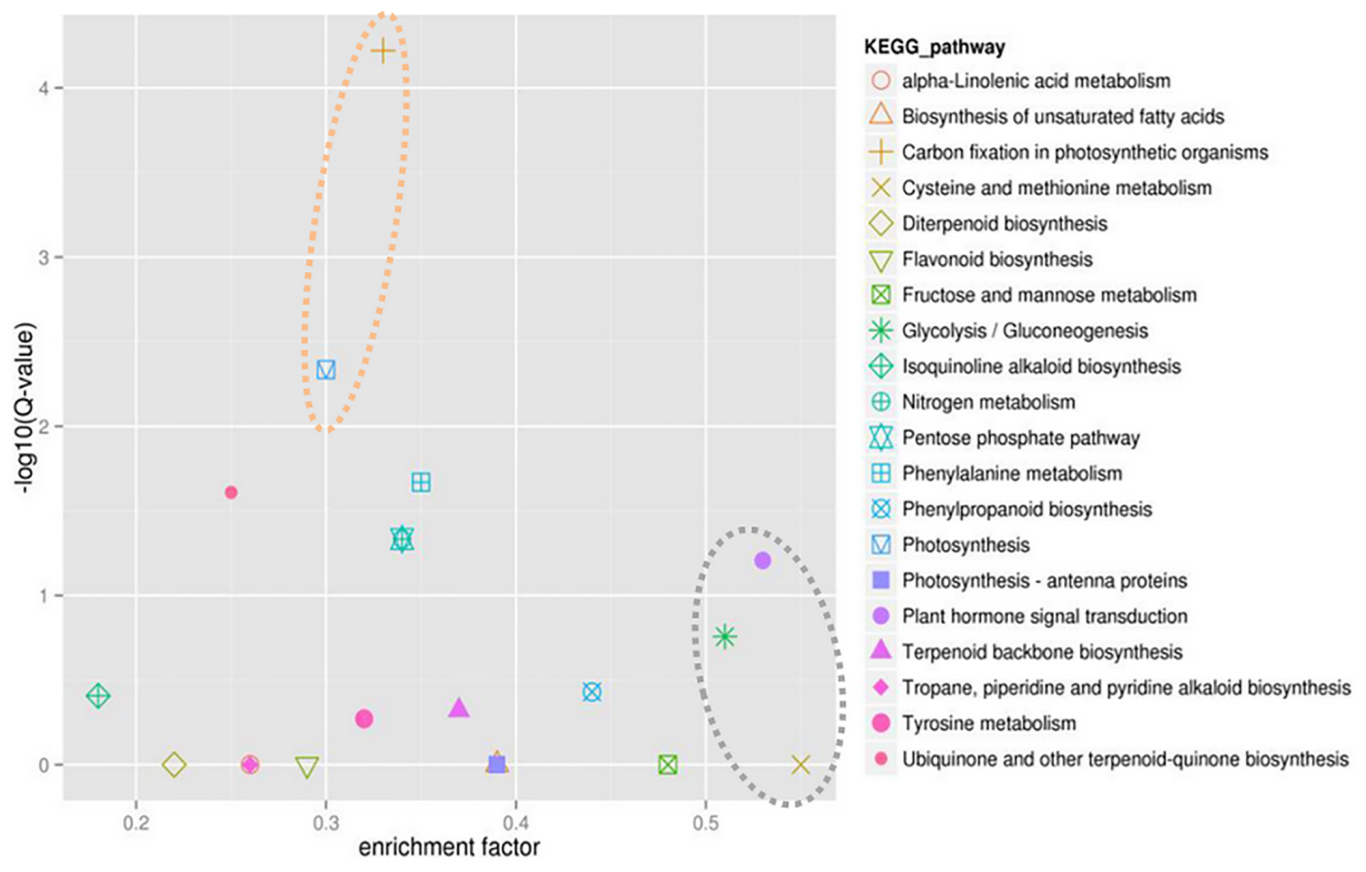
Top 20 significant KEGG enrichment pathways. Five pathways in cycles with dot line were the most significant and most enriched.

**Fig 7:**
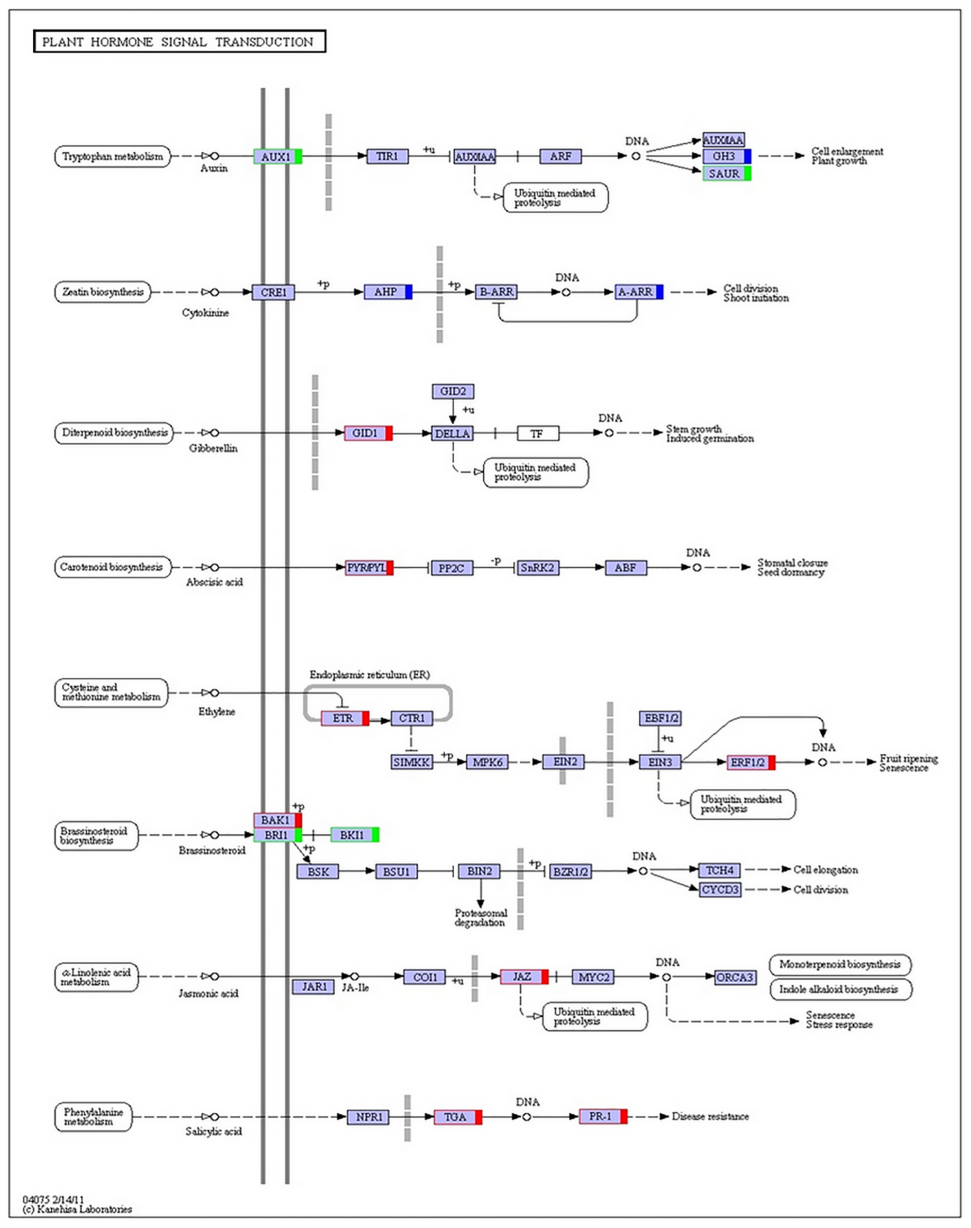
Genes encoding enzyme commission in plant hormone transduction pathway were down-regulated (green), up-regulated (red), and both up- and down-regulated (blue) in the transcriptome of susceptible mutant M14 compared to the resistant WT genotype.

Biological interpretation of the 2219 DEGs was also performed using KEGG pathway analysis. Top 20 pathways were analyzed based on the level of DEGs enrichment, suggesting the most significant DEGs were represented in the pathways of carbon fixation in photosynthetic organisms and photosynthesis, while the highest level of enriched DEGs were in the pathways of plant hormone signal transduction, glycolysis/gluconeogenesis, and cysteine and methionine metabolism (Fig 6). Four KEGG genes in glycolysis/gluconeogenesis pathway were significantly down-regulated and eight genes up-regulated in the susceptible M14 as shown in Fig 7.

Differential expression of genes involved in the transduction of plant hormones Several plant hormones are known to play roles in mediating plant defense response against pathogens. The major plant hormones involving stress response include auxins, gibberellins (GA), cytokinins (CK), abscisic acid (ABA), ethylene (ET), salicylic acid (SA), Jasmonates (JA), brassinosteroids (BR) and strigolactones [26]. In this study, activities of these plant hormones under pathogen initial challenge were showed in plant hormone transduction pathway (Fig 7). Auxins and BR were down-regulated while GA, ABA, ET, and SA were up-regulated, exception of CK without change, in the M14 compared to the WT. Transcripts for auxins and BR were also showed less expressed in the M14 but other hormone expression, such as GA, ABA, and ET, was increased (S1 Table).

### DEGs of defense-related genes

The most defense-related transcripts were surprised highly expressed in the susceptible M14 (88) than in the resistant WT (8), including pathogenesis-related proteins (PR proteins), NBS-LRR (R), LRR receptor-like proteins (LRR-RLK), and WRKY factors (S2 Table). The PR proteins, such as PR-1 (beta-1,3-glucanase), PR-4 (chitinase), PR-5 (thaumatin), and PR-12 (defensin), were significantly up-regulated in the susceptible M14, indicating they were quickly induced by pathogen infection. The most significantly expressed PR was thaumatin protein that was identical to the *AdTLP* protein resistant to LLS in the species *A*. *diogoi* [27]. Both susceptible and resistant genotypes were growing in the nature field condition; therefore, they could be infected by a number of pathogens.

### DEGs of photosynthesis

Unlike defense-related transcripts increased, all transcripts (48) related to photosynthesis were significantly decreased, such as photosystem II proteins (Psb), chlorophyll a-b binding proteins, photosystem repair proteins, ferredoxin-1, chloroplast movement proteins (CHUP1), and sedcheptulose-1,7-bisphosphatase (SBPase) except that Ferridoxin-3, chloroplastic was up-regulated in the susceptible genotype (S3 Table). Low level of these transcripts would affect plant photosynthesis and growth development.

### Confirmation of RNA sequencing data by qRT-PCR analysis

Quantitative real-time PCR (qPCR) was performed on selected genes with different expression levels and functional assignments to demonstrate the utility of RNA-Seq for gene expression profiles of defense responses (Table 3). In the present study, six of the identified DEGs were selected based on their association with diseases resistance response in peanut. The result was consistent with the annotation of these DEGs. The expression levels of all six of the selected DEGs exhibited up regulation in the mutant than its wild type.

**Table 3:** Confirmation of RNA seq data by qRT-PCR analysis.

Names	ID	RNASeq		qRT-PCR	
		log2 Fold Change		log2 Fold Change	
PR1-1	BMK.13440	4.596565	Up	7.994014	Up
PR1-2	BMK.41453	8.035185	Up	8.106013	Up
GID1	BMK.43270	3.7611319	Up	2.72465	Up
2.6.1.5	BMK.43468	4.1285357	Up	0.722466	Up
1.11.1.7–1	BMK.39352	3.5306467	Up	3.528571	Up
TGA2	BMK.42295	3.0059711	Up	0.201634	Up

## Discussion

The mechanism of plants response to the challenge by pathogens is complex and requires activating integrated pathways to defend pathogen attacks. One of such responses is to activate an inducible protein-based defense system that includes 17 families of pathogenesis-related (PR) protein termed PR-1 to PR-17 [28, 29]. Among PR proteins, proteins of PR-5 share significant amino acid homology to thaumatins, a sweet-tasting protein from the West African shrub, and are thus referred to as thaumatin-like protein (TLP) [30, 29]. Thaumatin proteins are considered as a prototype for a pathogen-response protein domain and have been studied extensively in plants for their antifungal properties [31–34]. A number of studies showed that PR-5 is involved in inhibition of hyphal growth and sporulation by various fungi by changing fungal cell permeability, disrupting cell wall structure, and perturbing fungal membrance structure [30], although their precise role is unknown. In the present study, PR proteins including PR1, PR-4, PR-5, and PR-12 were significantly up-regulated in the peanut susceptible genotype M14, particularly, transcripts of PR-5 (thaumatin) were highly expressed compared to the resistant WT. PR-5 transcripts identified in this study were identical to the *AdTLP* protein identified in *A*. *diogoi* by [27] (S2 Table). PR proteins rapidly induced at the earlier time point after fungal infection in susceptible plant while only marginal increase in resistant plants were noticed in European plum [35]. Higher level of defense-related proteins was expressed in the susceptible genotype M14, but it was eventually susceptible to pathogens. This phenomenon suggested there is other mechanism involved to reduce the capacity of the host defense to the challenge by pathogen.

The observation of significant decreased expression of DEGs in photosynthesis in the susceptible genotype prompted our interest to those DEGs. All 48, except one, genes related to photosynthesis were suppressed, including SBPase and chlorophyll a-b binding proteins, which may cause the host plant losing energy and weak development. It is believed that a low level of SBPase could reduce capacity to undergo photosynthesis, and less chlorophyll a-b binding proteins expressed also resulted in lack of vigor in plant development because they cannot capture and deliver excitation energy to photosystems. Several studies have showed that the relationship between photosynthesis and visual disease severity is related to the interaction between host and pathogen [36, 37]. A study showed that rice chloroplast protein PsbP interacts with a virus disease-specific protein and involved in the development of disease symptoms [38]. Down-regulation of genes related to photosynthesis strongly associated with disease symptoms was also reported by [39] that the chloroplast translation machinery was impaired by pathogen and failed in the electron transport that provides energy for photosynthesis. Taken together, it seems that expression of chloroplast genes was inhibited by elicitor generated by fungus in the susceptible plant.

Plant hormones serve as the key endogenous factors in mediating plant stress response [26]. In this study, plant hormones ABA, ET, JA, and SA were up-regulated under pathogen initial challenge in the susceptible M4 compared to the resistant WT. These hormones are known to play major roles in mediating plant defense response against pathogens and abiotic stress [26, 40]. Role of ABA in plant defense response is typically responsible for plant defense response against abiotic stresses. While as the levels of SA, JA and ET increased, these hormones play major roles in response to biotic stress conditions [41]. These increased hormones in the susceptible M14 provided evidence that were related to the high level of inducible protein-based defense system, particularly SA induced a high level of PR-1 in this study. However, hormones auxins and BR were decreased in the susceptible M14 compared to the resistant WT. Because auxins and BR are response to cell division and plant growth, reduced levels of auxins and BR may result in a less vigor plant and lead to a susceptibility in the M14.

WRKY transcription factors belong to a multigene family comprising over 75 members [42]. WRKY transcription factors not only participate in regulating plant defense to abiotic and biotic stressors [43] but also play key roles in regulation of developmentally programmed leaf senescence [44, 45]. Leaf senescence occurs in an age-dependent manner [46] but also can be genetic regulation triggered by various environmental factors, such as abiotic and biotic stress [47]. For instance, WRKY53 was identified as a positive senescence regulator [48], while WRKY70 could act as a negative regulator of senescence [49]. In this study, 22 WRKY transcription factors were significantly up-regulated in the susceptible genotype. These WRKY factors might involve in leaf senescence and accelerate forming disease symptoms. Leaf sencescence begins with chloroplast dismantling followed by catabolism of macromolecules such as chlorophyll, proteins, lipids, and RNA [45]. Increased WRKY factors may coordinate with low expression level of photosynthetic genes in the susceptible M14 to preclude the accumulation of PRs at the infection site and associated with disease symptom development. Because functional roles of WRKY proteins are involved in several cell death-related processes, including leaf senescence and pathogen defense, the complex network of regulation both in pathogen defense and leaf senescence would be further explored to understand how WRKY factors are involved in both defense to pathogens and leaf senescence.

The resistant genotype Yuanza 9102 was selected from an interspecific cross, where species *A*. *diogoi* was used as a parent. We identified several differentially expressed genes displaying an 8.5-fold (log2FC, *p* < 0.001) higher expression in the susceptible M14 as compared with the WT. Those DEGs shared high homology to thaumatin-like proteins previously identified in the species *A*. *diogoi*, that played key role in defense to LLS pathogen in peanut [11, 27]. Our resultant data might suggest that the resistant genes identified in this study were descending from the wild species *A*. *diogoi*. However, the number of up-regulated DEGs related to pathogenesis-related proteins in the susceptible M14 was markedly more than in the resistant WT by fungal infection, indicating that there was a hypersensitive reaction to pathogen infection in the mutant M14 at the earlier time point. The mutant M14 was finally susceptible to the LLS, which may involve integrated pathways that negatively mediated the defense responses and resulted in a failure to defense after pathogen infection. In this study, differentially expressed genes identified at one time point could not explain the dynamic changes in the level of expressed pathogenesis-related proteins in both susceptible M14 and resistant WT genotypes. Nevertheless, this study provides a significant transcriptome resource in leaves of peanut plant response to fungal infection. Photosynthetic genes, phytohormones, and WRKY factors were differentially expressed but coordinated in response to fungal infection between susceptible and resistant genotypes. In the susceptible M14, on one hand, some hormones mediating biotic stress response were up-regulated to increase the level of inducible defense-related proteins with pathogen infection. On the other hand, suppressed other hormones related to cell elongation and division and chloroplast genes inhibited plant growth and photosynthesis, which eventually result in disease symptoms though PRs were quickly expressed at the beginning time point of infection.

## Conclusions

RNA-Seq analysis is a comprehensive and high-throughput approach used to screen candidate genes and predict gene function. The availability of RNA-Seq has allowed us to acquire large-scale transcriptional changes at a genome-wide level. Huge amounts of transcriptomic data obtained from RNA-Seq analyses of various plant species susceptible to disease have enabled us to carry out comparisons of disease susceptible expression profiles of different plant species. The results obtained in this study showed that susceptible genotype initially generates higher level of pathogenesis-related proteins induced by plant hormones triggered with pathogen infection but finally becomes susceptible. It may involve other mechanism that lessen expression of defense to pathogen challenge, such as genes related to photosynthesis and plant hormones coordinated with WRKY factors reduced the capacity of photosynthesis, plant growth and leaded to symptoms in leaves. Overall, this study provides a basic foundation for further analyzing functional genes involving in the susceptible mechanism, which would ultimately allow us to gain insight into the interaction of plant host and pathogen.

## Supporting information

S1 Fig Difference of three phenotypic traits between M14 and WT. (TIF)

S1 Table List of expressed hormone transcripts. (DOCX)

S2 Table Characterization of defense-related unigenes annotated by Swissprot under fungal infection. (DOCX)

S3 Table Photosynthetic related unigenes annotated by Swissprot. (DOCX)
