# Supplementary figures and images for: Differential gene expression in leaf tissues between mutant and wild-type genotypes response to late leaf spot in peanut (Arachis hypogaea L.)
Source: PLoS One. 2017 Aug 25;12(8):e0183428. doi: 10.1371/journal.pone.0183428 (PMC5571927; doi:10.1371/journal.pone.0183428)

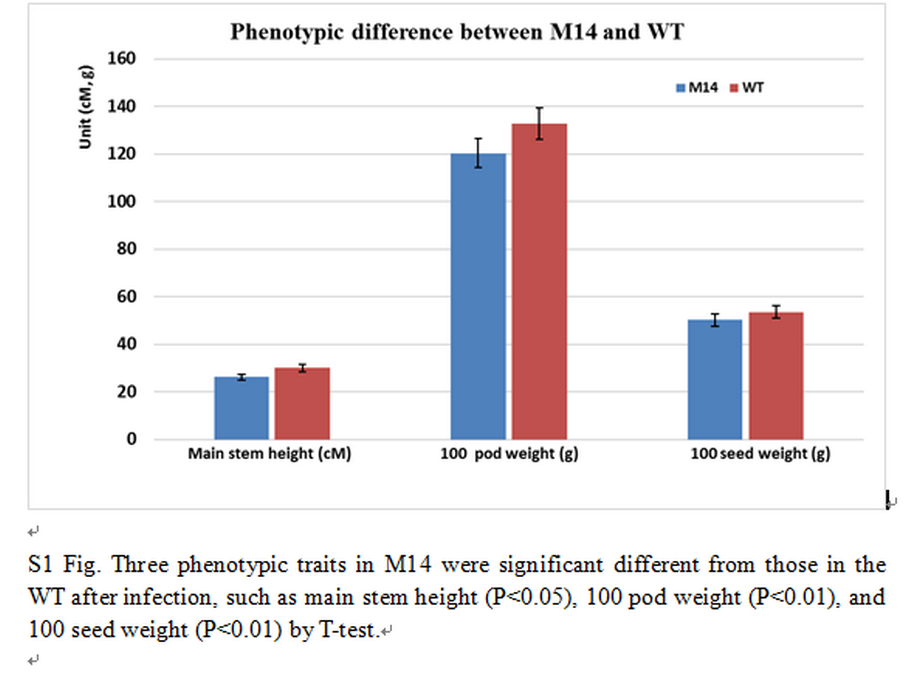

Supplement: S1 Fig — (TIF) [file pone.0183428.s001.tif]
