# Supplementary material for: Differential gene expression in leaf tissues between mutant and wild-type genotypes response to late leaf spot in peanut (Arachis hypogaea L.)
Source: PLoS One. 2017 Aug 25;12(8):e0183428. doi: 10.1371/journal.pone.0183428 (PMC5571927; doi:10.1371/journal.pone.0183428)
Supplement: S1 Table — (DOCX) [file pone.0183428.s002.docx]

**S1 Table. List of expressed hormone transcripts**

| **#ID** | **FDR** | **log2FC** | **regulated** | **Swissprot_annotation** |
| --- | --- | --- | --- | --- |
| BMK.18921 | 0.000301 | 3.591839 | up | Indole-3-acetic acid-amido synthetase |
| BMK.33460 | 5.52E-05 | 1.986777 | up | Abscisic acid receptor PYL4 |
| BMK.39363 | 5.00E-10 | 1.673983 | up | Ethylene-responsive transcription factor 1B |
| BMK.39452 | 1.98E-09 | 1.578093 | up | Ethylene-responsive transcription factor 15 |
| BMK.43011 | 4.15E-05 | 1.973389 | up | Indole-3-acetic acid-amido synthetase |
| BMK.43269 | 0 | 3.251149 | up | Gibberellin receptor GID1B |
| BMK.43270 | 0 | 3.761132 | up | Gibberellin receptor GID1B |
| BMK.44844 | 9.68E-05 | 1.092452 | up | Ethylene response sensor 1 |
| BMK.50321 | 5.34E-05 | 1.957701 | up | Ethylene receptor 2 |
| BMK.50322 | 1.11E-15 | 2.441481 | up | Ethylene receptor 2 |
| BMK.53073 | 3.48E-05 | 1.652725 | up | Brassinosteroid insensitive 1 -associated receptor |
| BMK.13207 | 2.90E-08 | -1.6831 | down | indole-3-acetic acid-amido synthetase |
| BMK.27041 | 1.11E-09 | -3.46477 | down | Auxin-induced protein |
| BMK.27042 | 8.10E-12 | -3.69354 | down | Auxin-induced protein |
| BMK.34581 | 3.41E-10 | -1.49753 | down | Auxin-responsive protein IAA14 |
| BMK.35915 | 0.000188 | -1.8339 | down | Auxin-induced protein X15 |
| BMK.36873 | 0 | -2.41068 | down | Auxin-induced protein 22C |
| BMK.37767 | 1.31E-08 | -1.37454 | down | Auxin-induced protein 22B |
| BMK.41070 | 1.48E-06 | -1.26181 | down | Auxin transporter-like protein 2 |
| BMK.53930 | 0.000302 | -1.02772 | down | Auxin response factor 9 |
| BMK.43540 | 1.55E-13 | -1.79173 | down | Auxin-responsive protein IAA20 |
| BMK.53232 | 6.47E-06 | -1.32202 | down | Brassinosteroid LRR receptor kinase |
