# Supplementary material for: Differential gene expression in leaf tissues between mutant and wild-type genotypes response to late leaf spot in peanut (Arachis hypogaea L.)
Source: PLoS One. 2017 Aug 25;12(8):e0183428. doi: 10.1371/journal.pone.0183428 (PMC5571927; doi:10.1371/journal.pone.0183428)
Supplement: S2 Table — (DOCX) [file pone.0183428.s003.docx]

**S2 Table. Characterization of defense-related unigenes annotated by Swissprot under fungal infection**

| **#ID** | **FDR** | **log2FC** | **regulated** | **Swissprot annotation** |
| --- | --- | --- | --- | --- |
| BMK.57337 | 0 | 8.494055 | up | Thaumatin-like protein AdTLP |
| BMK.14425 | 0 | 8.101273 | up | Thaumatin-like protein 1 |
| BMK.41453 | 0 | 8.035185 | up | Pathogenesis-related protein 1C |
| BMK.31888 | 0 | 7.212849 | up | Glucan endo-1,3-beta-glucosidase, acidic isoform PR-N |
| BMK.21363 | 0 | 6.844622 | up | Probable WRKY transcription factor 70 |
| BMK.14424 | 0 | 6.7471 | up | Glucan endo-1,3-beta-glucosidase |
| BMK.43521 | 0 | 6.682251 | up | Probable WRKY transcription factor 50 |
| BMK.34039 | 0 | 5.799856 | up | Wound-induced protein WIN2 |
| BMK.45125 | 0 | 5.535805 | up | Probable WRKY transcription factor 40 |
| BMK.19141 | 3.40E-11 | 5.413201 | up | Glucan endo-1,3-beta-glucosidase |
| BMK.42194 | 0 | 5.409979 | up | Glucan endo-1,3-beta-glucosidase |
| BMK.25830 | 0 | 5.23419 | up | Wound-induced protein WIN1 |
| BMK.36009 | 0 | 4.816723 | up | Thaumatin-like protein |
| BMK.19714 | 0 | 4.756851 | up | Disease resistance protein RPM1 |
| BMK.14033 | 0 | 4.718317 | up | Thaumatin-like protein 1a |
| BMK.41356 | 0 | 4.637049 | up | Probable LRR receptor-like serine/threonine-protein kinase |
| BMK.13440 | 7.15E-06 | 4.596565 | up | Pathogenesis-related protein 1 |
| BMK.13363 | 3.00E-09 | 4.440509 | up | Defensin-like protein |
| BMK.45434 | 0 | 4.324847 | up | Probable WRKY transcription factor 50 |
| BMK.37724 | 0 | 4.241552 | up | Pathogenesis-related protein 1C |
| BMK.43468 | 0 | 4.1285357 | up | Probable aminotransferase TAT2 |
| BMK.34835 | 1.66E-09 | 4.062059 | up | Probable WRKY transcription factor 75 |
| BMK.40073 | 0 | 4.059299 | up | Protein P21 |
| BMK.52739 | 0 | 4.015524 | up | Probable WRKY transcription factor 75 |
| BMK.3247 | 9.42E-08 | 3.822917 | up | Disease resistance protein RPM1 |
| BMK.43270 | 0 | 3.7611319 | up | Gibberellin receptor GID1B-like |
| BMK.39352 | 0 | 3.5306467 | up | Peroxidase 55-like |
| BMK.19715 | 0 | 3.311852 | up | Disease resistance protein RPM1 |
| BMK.40920 | 0 | 3.073865 | up | Probable WRKY transcription factor 50 |
| BMK.35588 | 1.59E-09 | 3.050718 | up | Probable WRKY transcription factor 28 |
| BMK.42295 | 0 | 3.0059711 | up | Transcription factor HBP-1b(c38)-like |
| BMK.40397 | 2.16E-05 | 2.702839 | up | Probable LRR receptor-like serine/threonine-protein kinase |
| BMK.55970 | 0 | 2.681602 | up | LRR receptor-like serine/threonine-protein kinase *FLS2* |
| BMK.54826 | 3.32E-08 | 2.673894 | up | Probable WRKY transcription factor 40 |
| BMK.37138 | 0 | 2.603439 | up | Disease resistance response protein DRRG49-C |
| BMK.29113 | 4.35E-06 | 2.534639 | up | Probable WRKY transcription factor 19 |
| BMK.40419 | 0 | 2.511463 | up | Probable WRKY transcription factor 40 |
| BMK.31802 | 0.000379 | 2.486568 | up | Probable LRR receptor-like serine/threonine-protein kinase |
| BMK.42886 | 0 | 2.371886 | up | TMV resistance protein N |
| BMK.26475 | 0.002276 | 2.332833 | up | Leucine-rich repeat receptor-like protein kinase PXL2 |
| BMK.15587 | 0 | 2.291091 | up | Defensin-like protein |
| BMK.51967 | 5.43E-10 | 2.174692 | up | Probably inactive leucine-rich repeat receptor-like protein kinase |
| BMK.55968 | 6.00E-05 | 2.134632 | up | LRR receptor-like serine/threonine-protein kinase FLS2 |
| BMK.55034 | 3.45E-10 | 2.08648 | up | Probable LRR receptor-like serine/threonine-protein kinase |
| BMK.36097 | 1.11E-16 | 2.048582 | up | Pathogenesis-related protein STH-2 |
| BMK.53001 | 0 | 2.043159 | up | Receptor-like protein kinase HAIKU2 |
| BMK.35812 | 7.55E-08 | 2.04085 | up | LRR receptor-like serine/threonine-protein kinase ERECTA |
| BMK.34614 | 0.000629 | 2.015951 | up | Probable LRR receptor-like serine/threonine-protein kinase |
| BMK.43057 | 1.44E-11 | 1.994537 | up | Probable WRKY transcription factor 15 |
| BMK.49016 | 5.42E-10 | 1.963731 | up | Leucine-rich repeat receptor-like serine/threonine-protein kinase |
| BMK.41957 | 1.21E-08 | 1.918297 | up | WRKY transcription factor 6 |
| BMK.28296 | 7.80E-09 | 1.88143 | up | Probable LRR receptor-like serine/threonine-protein kinase |
| BMK.40395 | 0.000312 | 1.842836 | up | Probable LRR receptor-like serine/threonine-protein kinase |
| BMK.54828 | 0.000401 | 1.825654 | up | Probable WRKY transcription factor 40 |
| BMK.43056 | 2.43E-11 | 1.771208 | up | Probable WRKY transcription factor 15 |
| BMK.56229 | 1.29E-09 | 1.737424 | up | Disease resistance protein |
| BMK.45029 | 8.69E-10 | 1.725287 | up | Probable WRKY transcription factor 72 |
| BMK.33477 | 0.004883 | 1.698595 | up | TMV resistance protein *N* |
| BMK.41096 | 6.00E-10 | 1.692117 | up | Probable WRKY transcription factor 69 |
| BMK.43754 | 3.16E-05 | 1.548003 | up | Probable WRKY transcription factor 48 |
| BMK.30833 | 1.48E-05 | 1.54004 | up | Putative disease resistance *RPP13*-like protein 1 |
| BMK.44645 | 2.08E-07 | 1.496439 | up | Probable WRKY transcription factor 11 |
| BMK.45760 | 0.008119 | 1.467409 | up | Probable LRR receptor-like serine/threonine-protein kinase |
| BMK.37554 | 1.75E-07 | 1.430647 | up | Probable WRKY transcription factor 50 |
| BMK.48802 | 4.89E-08 | 1.399371 | up | Probable LRR receptor-like serine/threonine-protein kinase |
| BMK.15014 | 0.001275 | 1.373462 | up | Putative disease resistance protein |
| BMK.14573 | 1.04E-07 | 1.349713 | up | Probable disease resistance protein |
| BMK.41737 | 2.04E-07 | 1.324631 | up | Pathogen-related protein |
| BMK.51592 | 0.002983 | 1.288971 | up | Disease resistance protein RPS5 |
| BMK.54965 | 8.27E-05 | 1.276357 | up | Disease resistance protein |
| BMK.53881 | 4.10E-06 | 1.275849 | up | TMV resistance protein N |
| BMK.50586 | 0.000609 | 1.257044 | up | LRR receptor-like serine/threonine-protein kinase GSO1 |
| BMK.27125 | 0.000149 | 1.245309 | up | Probable WRKY transcription factor 30 |
| BMK.56672 | 7.70E-06 | 1.239485 | up | Probable disease resistance protein |
| BMK.56441 | 0.000267 | 1.217364 | up | Disease resistance protein |
| BMK.55712 | 4.68E-06 | 1.214533 | up | LRR receptor-like serine/threonine-protein kinase GSO1 |
| BMK.46215 | 9.70E-05 | 1.204714 | up | Putative disease resistance protein |
| BMK.41826 | 0.002463 | 1.195975 | up | Probable leucine-rich repeat receptor-like protein kinase |
| BMK.43793 | 4.90E-05 | 1.178972 | up | Probably inactive leucine-rich repeat receptor-like protein kinase |
| BMK.48801 | 0.00154 | 1.176602 | up | Probable LRR receptor-like serine/threonine-protein kinase |
| BMK.43419 | 1.27E-05 | 1.172972 | up | Leucine-rich repeat receptor-like serine/threonine/tyrosine-protein kinase  SOBIR1 |
| BMK.42649 | 5.52E-05 | 1.150405 | up | TMV resistance protein N |
| BMK.49867 | 9.32E-05 | 1.143957 | up | Putative disease resistance RPP13-like protein 1 |
| BMK.48227 | 0.000398 | 1.12648 | up | LRR receptor-like serine/threonine-protein kinase EFR |
| BMK.43794 | 0.001107 | 1.119997 | up | Probably inactive leucine-rich repeat receptor-like protein kinase |
| BMK.53952 | 0.002488 | 1.11993 | up | Probable leucine-rich repeat receptor-like serine/threonine-protein kinase |
| BMK.55832 | 0.000566 | 1.074338 | up | Leucine-rich repeat receptor-like tyrosine-protein kinase |
| BMK.56365 | 0.000507 | 1.042984 | up | Probable disease resistance protein RDL6 |
| BMK.54507 | 0.000334 | 1.038935 | up | LRR repeats and ubiquitin-like domain-containing protein |
| BMK.33929 | 0.000359 | 1.035646 | up | Putative late blight resistance protein homolog R1A-6 |
| BMK.48098 | 0.000606 | 1.016036 | up | WRKY transcription factor 6 |
| BMK.34215 | 0.00421 | 1.00554 | up | Probable disease resistance protein |
| BMK.35536 | 0.000615 | -1.04828 | down | LRR receptor-like serine/threonine-protein kinase GSO2 |
| BMK.21173 | 0.000129 | -1.0579 | down | Probable salt tolerance-like protein |
| BMK.47708 | 0.000291 | -1.16332 | down | Probable inactive leucine-rich repeat receptor-like protein kinase |
| BMK.34090 | 7.41E-06 | -1.20864 | down | Leucine-rich repeat receptor-like serine/threonine-protein kinase BAM1 |
| BMK.39535 | 0.00116 | -1.27639 | down | Putative disease resistance protein RGA4 |
| BMK.41549 | 0.000351 | -1.44743 | down | LRR receptor-like serine/threonine-protein kinase RCH1 |
| BMK.32880 | 0.000451 | -1.54018 | down | Thaumatin-like protein 1 |
| BMK.30682 | 4.57E-05 | -1.59206 | down | Thaumatin-like protein 1 |
