# Supplementary material for: Differential gene expression in leaf tissues between mutant and wild-type genotypes response to late leaf spot in peanut (Arachis hypogaea L.)
Source: PLoS One. 2017 Aug 25;12(8):e0183428. doi: 10.1371/journal.pone.0183428 (PMC5571927; doi:10.1371/journal.pone.0183428)
Supplement: S3 Table — (DOCX) [file pone.0183428.s004.docx]

**S3 Table．Photosynthetic related unigenes annotated by Swissprot**

| **#ID** | **FDR** | **log2FC** | **regulated** | **Swissprot_annotation** |
| --- | --- | --- | --- | --- |
| BMK.29087 | 4.43E-10 | 1.604092 | up | Ferredoxin-3, chloroplastic |
| BMK.28514 | 0.000543 | -1.00085 | down | Photosystem II repair protein PSB27-H1, chloroplastic |
| BMK.29277 | 0.000652 | -1.00703 | down | ATP synthase subunit b&apos;, chloroplastic |
| BMK.34561 | 0.00043 | -1.01026 | down | PsbQ-like protein 2, chloroplastic |
| BMK.48514 | 0.000435 | -1.02136 | down | Transcriptional corepressor SEUSS |
| BMK.37480 | 0.000377 | -1.03987 | down | Photosystem II protein psbY-2, chloroplastic |
| BMK.37542 | 0.001234 | -1.04151 | down | Chlorophyll a-b binding protein 7, chloroplastic |
| BMK.43869 | 0.000324 | -1.05708 | down | PsbP-like protein 1, chloroplastic |
| BMK.54231 | 0.000126 | -1.05948 | down | Myosin-2 |
| BMK.39101 | 0.000108 | -1.06447 | down | Putative glucose-6-phosphate 1-epimerase |
| BMK.55197 | 0.000265 | -1.06648 | down | Inositol-3-phosphate synthase |
| BMK.44683 | 0.000132 | -1.06693 | down | Violaxanthin de-epoxidase, chloroplastic |
| BMK.48441 | 6.48E-05 | -1.10125 | down | Serine/threonine-protein kinase STN8, chloroplastic |
| BMK.43458 | 0.000485 | -1.10712 | down | Photosystem II D1 precursor processing protein PSB27-H2, chloroplastic |
| BMK.51382 | 7.01E-05 | -1.11225 | down | ATP synthase gamma chain, chloroplastic |
| BMK.39050 | 0.000106 | -1.12203 | down | Ferredoxin--NADP reductase, leaf isozyme 1, chloroplastic |
| BMK.37453 | 2.00E-05 | -1.12785 | down | Phospholipid hydroperoxide glutathione peroxidase, chloroplastic |
| BMK.40971 | 4.33E-05 | -1.12977 | down | Probable lipid desaturase ADS3.2, chloroplastic |
| BMK.38343 | 3.77E-05 | -1.13843 | down | Sedoheptulose-1,7-bisphosphatase, chloroplastic |
| BMK.44125 | 1.46E-05 | -1.14174 | down | Chlorophyllide a oxygenase, chloroplastic |
| BMK.14453 | 2.90E-05 | -1.14299 | down | Rho guanine nucleotide exchange factor 8 |
| BMK.40808 | 9.68E-06 | -1.16724 | down | Chlorophyll a-b binding protein P4, chloroplastic |
| BMK.57348 | 9.18E-06 | -1.18825 | down | Phosphoribulokinase, chloroplastic |
| BMK.34968 | 3.63E-06 | -1.19039 | down | PsbQ-like protein 1, chloroplastic |
| BMK.25671 | 3.76E-06 | -1.19627 | down | Photosystem I reaction center subunit N, chloroplastic |
| BMK.51237 | 4.09E-06 | -1.19971 | down | Protein CHUP1, chloroplastic |
| BMK.37247 | 8.71E-06 | -1.21425 | down | Photosystem II 22 kDa protein, chloroplastic |
| BMK.35621 | 8.50E-06 | -1.21828 | down | Chlorophyll a-b binding protein 215, chloroplastic |
| BMK.44181 | 4.62E-06 | -1.21842 | down | Pentatricopeptide repeat-containing protein |
| BMK.31711 | 1.54E-06 | -1.23479 | down | Chlorophyll a-b binding protein 13, chloroplastic |
| BMK.32049 | 3.09E-06 | -1.23884 | down | Chlorophyll a-b binding protein 8, chloroplastic |
| BMK.47978 | 4.62E-07 | -1.26087 | down | PsbB mRNA maturation factor Mbb1, chloroplastic |
| BMK.32006 | 5.33E-07 | -1.27518 | down | Photosystem I reaction center subunit psaK, chloroplastic |
| BMK.36914 | 2.88E-07 | -1.28185 | down | Photosystem I reaction center subunit IV A isoform 2 |
| BMK.33309 | 1.58E-07 | -1.30541 | down | PsbP-like protein 2, chloroplastic |
| BMK.53966 | 1.28E-06 | -1.30832 | down | Pentatricopeptide repeat-containing protein |
| BMK.54369 | 9.02E-08 | -1.31899 | down | Phototropin-2 |
| BMK.40857 | 8.08E-08 | -1.33264 | down | Glycerate dehydrogenase |
| BMK.31593 | 3.47E-08 | -1.35374 | down | Photosystem II reaction center PSB28 protein, chloroplastic |
| BMK.55035 | 7.51E-08 | -1.36071 | down | Tetrapyrrole-binding protein, chloroplastic |
| BMK.29329 | 5.35E-08 | -1.36631 | down | Chlorophyll a-b binding protein CP26, chloroplastic |
| BMK.32946 | 9.77E-09 | -1.38351 | down | Thylakoid membrane phosphoprotein 14 kDa, chloroplastic |
| BMK.29279 | 3.82E-09 | -1.41963 | down | Chlorophyll a-b binding protein 13, chloroplastic |
| BMK.35353 | 2.21E-09 | -1.43418 | down | Magnesium-protoporphyrin IX monomethyl ester [oxidative] cyclase,  chloroplastic |
| BMK.38269 | 1.52E-09 | -1.44421 | down | Fructose-1,6-bisphosphatase, chloroplastic |
| BMK.32410 | 3.58E-10 | -1.49059 | down | Ferredoxin-1, chloroplastic |
| BMK.48915 | 4.35E-11 | -1.56544 | down | RNA polymerase sigma factor sigE, chloroplastic/mitochondrial |
| BMK.30088 | 1.32E-13 | -1.71807 | down | Protein PROTON GRADIENT REGULATION 5, chloroplastic |
